# Inner Branched Endovascular Repair of a Post-Type B Dissection Aneurysm Previously Treated With a Nellix in Both Lumens: Making the Most of a Complex Situation

**DOI:** 10.1016/j.ejvsvf.2026.01.007

**Published:** 2026-02-05

**Authors:** Talje M. Fokkema, Wajdi Alrawi, Håkan Åstrand, Robert C. Lind

**Affiliations:** a Department of Vascular Surgery, Ryhov Country Hospital, Jönköping, Sweden; b Department of Thoracic and Vascular Surgery, Linköping University Hospital, Linköping, Sweden; c Department of Health, Medicine and Caring Sciences, Linköping University, Linköping, Sweden

**Keywords:** Aneurysm, Chronic type B aortic dissection, Complex anatomy, Inner branches

## Abstract

**Introduction:**

Treatment of chronic type B aortic dissection associated aneurysms where key vessels originate from both the true and false lumens is technically challenging. Further aneurysm formation may require new interventions after initial treatment. Innovative endovascular techniques can lead to successful treatment.

**Report:**

A 74 year old man with a type B dissection was treated with an urgent femorofemoral crossover bypass (in 2001) and surgical graft in the thoracic aorta (in 2003). Due to the combination of a chronic type B dissection and a right sided common iliac artery aneurysm, he was subsequently treated with two separate Nellix endoprotheses (Endologix Inc., Irvine, CA, USA) in the infrarenal aorta. The Nellix endoprotheses were deployed in the true and the false lumens (in 2015). Eight years later, he required another intervention due of progressive enlargement of the thoracic aorta between the surgical graft and Nellix endoprotheses. The chosen treatment was a customised two inner branch thoracic endovascular aortic repair (TEVAR) and four inner branch endovascular aortic repairs, in combination with long Viabahn stent grafts from the TEVAR to the old Nellix endoprothesis in the false lumen (in 2021). All stent grafts were patent at three years of follow up.

**Discussion:**

This case report presents a patient in whom former techniques were used as an initial treatment for a chronic type B dissection with aneurysm formation complicating further treatment. This report highlights what can be achieved with careful planning in combination with new available devices, resulting in a good individual outcome.

## Highlights

• Chronic type B aortic dissection aneurysms can be challenging to treat
• Inner branched endografts can be used successfully in a small aortic lumen
• Very complex aortic anatomy can still be treated with endovascular techniques
• Outside of the box solutions are sometimes needed

## INTRODUCTION

Aortic related complications occur in 20–50% of patients with chronic type B aortic dissection (CTBAD),^1^ with 20–40% of patients developing aneurysms that warrant treatment.^2^ The expansion rate of CTBAD aneurysms is not well established but has been reported to be between 1 – 7 mm per year.^3^ In asymptomatic patients, the maximum CTBAD aneurysm diameter remains the most important indication for treatment, with current aortic guidelines recommending repair above 55 mm.^4^ CTBAD aneurysms can be challenging to treat, especially after previous aortic surgery. This case report presents a patient with a CTBAD aneurysm after previous open and endovascular aortic repairs, highlighting the versatility of inner branched endovascular aortic repair (iBEVAR).

## REPORT

**Figure 1:**
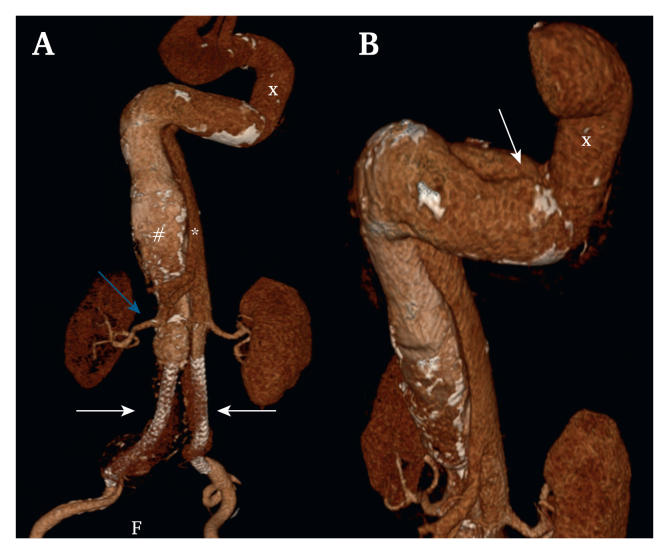
3D reconstruction showing the CTBAD aneurysm. (A) Residual chronic type B aortic dissection with aneurysm formation between the surgical graft (x) and Nellix endoprothesis (white arrows). The Nellix endoprotheses were previously placed in both the false (#) and true (∗) lumens. All renovisceral arteries, except for the right renal artery (yellow arrow), originate from the small true lumen. (B) The dissection membrane (white arrow) started just distal to the surgical graft (x) in the descending aorta.

A 74 year old man with a history of hypertension, activated protein C resistance, atrial fibrillation, and epigastric and groin hernias presented with an acute type B aortic dissection in 2001. All renovisceral vessels, except for the right renal artery (RRA), originated from the true lumen (TL). The right lower limb was perfused through the false lumen (FL) and left lower limb through the TL. Due to malperfusion of the left lower limb, urgent femorofemoral crossover bypass was performed. The bypass occluded after three years without symptoms. In 2003, the patient developed a CTBAD aneurysm of the descending aorta, which was treated with a surgical graft through a left sided thoracotomy where distal circulation to both the true and false lumens was preserved. In 2015, the patient developed a right common iliac artery (RCIA) aneurysm. Because the circulation to the lower limbs originated from different lumens, the RCIA aneurysm was treated with Nellix (Endologix, Inc., Irvine, CA, USA) endoprotheses. The right Nellix was placed through the FL, and the left Nellix through the TL. Both Nellix endoprostheses were deployed in the infrarenal aorta. Progressive aneurysmal degeneration occurred in the untreated dissected native aorta between the thoracic surgical graft and Nellix endoprotheses (Fig. 1). When a diameter >60 mm was reached in 2021, prophylactic endovascular repair was performed.

**Figure 2:**
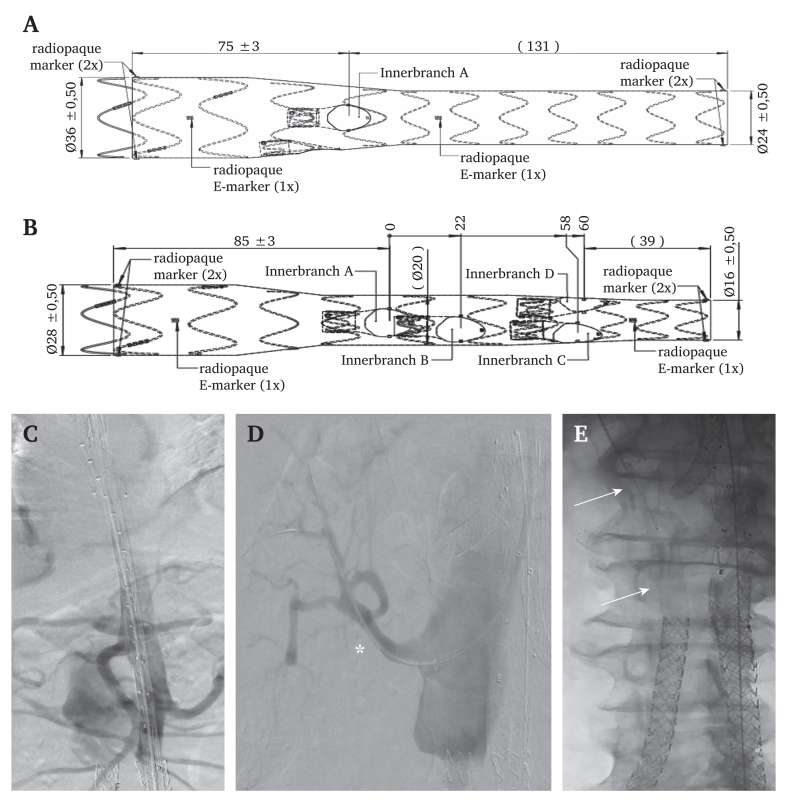
Schematic representation of the (A) custom made thoracic endograft and (B) inner branched endovascular aortic repair (iBEVAR). The thoracic endograft has two inner branches and four in the iBEVAR. (C) After placement of the thoracic endograft, the iBEVAR was placed in the tight true lumen. (D) The right renal artery (∗) was accessed through the dissection membrane. (E) Long Viabahn stent grafts (arrows) were placed from the branch in the thoracic endograft through the false lumen into the right sided Nellix, thereby preserving circulation to the right lower limb.

Endovascular repair was complicated by the Nellix endoprotheses in the false and true lumens. Proximal sealing was planned in the surgical graft in the descending aorta. In close cooperation with the manufacturer, two custom made inner branch devices (E-xtra Design Multibranch, Artivion, Kennesaw, GA, USA) were planned. The first was a thoracic endograft with an 8 mm branch, which was planned to be connected to the right Nellix through the FL, and a 6 mm perfusion branch to minimise the risk of spinal cord ischaemia. The second endograft was an iBEVAR, to be placed in the compressed TL (mean TL diameter 19 mm), with inner branches for the coeliac trunk (CT), superior mesenteric artery (SMA), RRA, and left renal artery (LRA; Fig. 2A and B). The CT, SMA, and LRA branches were planned at the proximal level of the target vessels, and the RRA branch at the level of a fenestration in the dissection membrane.

A two stage repair was planned to reduce the risk of spinal cord ischaemia and limit Xray time. Other preventive measures taken during the operation were mean arterial blood pressure >80 mmHg, active clotting time ≥300 seconds, and spinal fluid drainage.

Ultrasound guided retrograde access to the left axillary artery and left common femoral artery (CFA) was established. A 12 F Ansel introducer sheath (Cook Medical, Bloomington, IN, USA) was advanced through the axillary artery into the proximal descending aorta. An 8 F introducer sheath (Brite Tip, Cordis, Miami Lakes, FL, USA) was placed in the left CFA. Pre-closure with a single ProStyle Suture (Abbott, Abbott Park, IL, USA) was performed at both access sites. A through and through guidewire was established from the left CFA through the Nellix and TL to the axillary artery access site. The proximal end of the left Nellix was dilated to 14 mm using a PTA balloon. The 8 F introducer was removed and the thoracic endograft was advanced over the through and through guidewire into the surgical graft. The thoracic endograft was deployed with both inner branches above the dissection membrane. Distal extension with the iBEVAR, with judicious attention to rotation, through the narrow TL into the left Nellix was subsequently performed (Fig. 2C). During deployment, traction was maintained on the delivery system to counteract friction between the Nellix and outer sheath to avoid pushing the endograft up during each 4 mm deployment step, thereby ensuring correct longitudinal alignment.

The iBEVAR delivery system was removed and a 16 F Dryseal introducer sheath (W.L. Gore & Associates, Flagstaff, AZ, USA) placed through the left CFA. A 16 x 38 mm BeGraft Aortic (Bentley InnoMed, Hechingen, Germany) balloon expandable covered stent was then used to achieve an adequate distal seal. Both endografts were moulded using a compliant balloon in standard fashion. Hereafter, the 12 F Ansel introducer was advanced from the descending aorta into the iBEVAR. The inner branches and target vessels were sequentially accessed and bridging stent grafts deployed through an 8 F Destination introducer sheath (Terumo, Tokyo, Japan) placed coaxially through the 12 F Ansel. The CT was revascularised using two 10 mm Begraft+ (37 and 27 mm; Bentley InnoMed) balloon expandable covered stents: the SMA with an 8 x 57 mm Begraft+ and the LRA with an 7 x 37 mm Begraft+, which was extended with an 8 x 50 mm Viabahn self expanding stent graft (W.L. Gore & Associates). Lastly, the RRA, originating from the FL was accessed through the dissection membrane and revascularised using an 8 x 80 mm Covera self expanding stent graft (BD, Franklin Lakes, NJ, USA; Fig. 2D). The FL was then accessed through the 8 mm inner branch in the thoracic endograft and the right sided Nellix endoprothesis was catheterised. An 8 x 250 mm Viabahn was placed from the inner branch into the FL and thereafter extended into the Nellix with a 10 x 150 mm Viabahn (Fig. 2E). An 9 x 19 mm Omnilink balloon expandable stent (Abbott) was used to lock the proximal Viabahn in the inner branch. The perfusion branch was left open. Post-operative recovery was uneventful. The patient was placed on apixaban due to atrial fibrillation.

Six weeks later, planned angiography was performed under local anaesthesia revealing that the perfusion branch had occluded without neurological symptoms. A type 1b endoleak to the RCIA aneurysm and internal iliac artery (IIA) was successfully treated with endovascular plugs in the IIA and parallel to the distal end of the Nellix endoprothesis.

**Figure 3:**
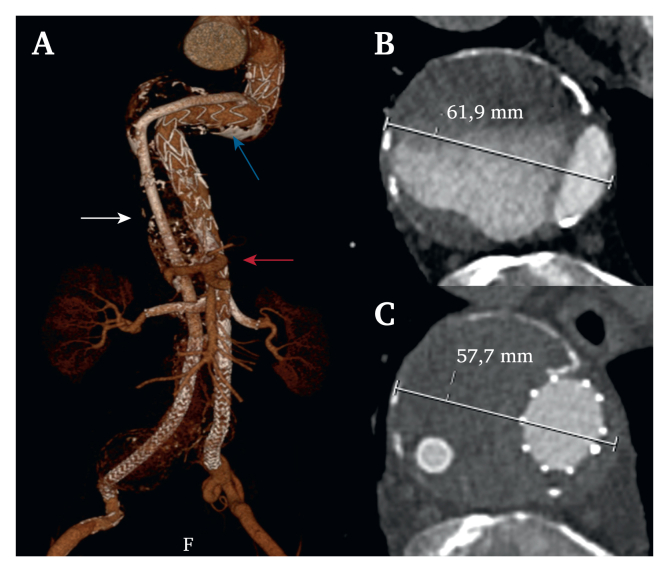
Three dimensional reconstruction at three year follow up showing the thoracic endograft (blue arrow) extended distally with the inner branched endovascular aortic repair (iBEVAR) (red arrow) through the true lumen to the left Nellix and the long Viabahn stent grafts through the false lumen to the right Nellix (white arrow). All renovisceral side branches are patent at three years. A reduction in aneurysm size is seen from (B) pre-operation to (C) three year follow up. Note that the iBEVAR has expanded the true lumen.

At three years, all endografts and target vessels remain patent, there are no endoleaks and the aneurysm has decreased from 62 mm to 58 mm (Fig. 3). Connections between the long 8 mm and 10 mm Viabahns, and 10 mm Viabahn and Nellix remain stable.

## DISCUSSION

CTBAD aneurysms can be challenging to treat, especially after previous aortic interventions and when target vessels originate from different lumens. This case report describes a CTBAD case previously treated by both open and endovascular techniques, where aneurysmal degradation necessitated further intervention. The presence of an occluded femorofemoral crossover bypass and Nellix endoprostheses in both the true and false lumens limited and complicated the endovascular options for repair. Repair was performed using two custom made inner branch endografts to preserve flow to the renovisceral vessels and lower limbs through the true and false lumens.

The use of commercially available off the shelf branched devices (e.g., Cook Medical's T-Branch and Artivion's E-nside) has previously been described in narrow anatomies, with promising results.^5^^,^^6^ The partial deployment technique of the T-branch in narrow anatomy can achieve high technical success^7^ but, in this case, outer branches would have led to longer bridging stent grafts in the narrow true lumen, increasing the risk of bridging stent graft compression. The proximal and distal diameters of both the T-branch (36 mm and 18 mm, respectively) and E-nside (smallest available 33 mm and 26 mm, respectively) were also deemed inappropriate compared with the true lumen and Nellix diameters.

A custom made fenestrated device (FEVAR) was considered but fenestration planning and alignment is challenging in small and compressed lumens. Furthermore, the RRA originated from the FL, requiring a long bridging stent and predisposing it to instability in FEVAR.^8^ Fenestrations and target vessels would need to have been cannulated before diameter reducing tie release, as this would have been impossible through the contralateral groin.

Custom made iBEVAR was chosen because inner branches are more forgiving in misalignment than fenestrations, thereby simplifying planning. Furthermore, target vessels do not need to be catheterised before complete deployment of the endograft and shorter bridging stent grafts can be used, reducing the risk of compression compared with off the shelf branched devices. Compared with FEVAR, iBEVAR offers theoretical advantages, including improved bridging stent graft mating and sealing.^9^ However, inner branches can lead to a reduction in residual lumen in the endoprothesis but this can now be limited by using semi branches.

iBEVAR is an emerging treatment modality that allows for the use of branches even in narrow anatomies. This can be especially useful in CTBAD aneurysms with thoraco-abdominal involvement where narrow lumens need to be accessed, and dissection membranes crossed.

Transcatheter electrosurgical septotomy might now be used in similar cases. A septotomy creates a single large channel by combining both lumens, creating more space and thereby potentially simplifying endograft design, deployment, and access to target vessels.^10^

In conclusion, iBEVAR represents a promising minimally invasive treatment option for CTBADs aneurysms. The versatility of a custom made iBEVAR allows it to preserve side branch perfusion, even in narrow anatomies, making it a valuable tool in the vascular surgeon's armamentarium, helping vascular surgeons make the most of complex situations.

## Funding

None.

## Conflicts of interest

R.L. receives speaker fees from Gore.

## References

[1] Winnerkvist A., Lockowandt U., Rasmussen E., Radegran K. (2006). A prospective study of medically treated acute type B aortic dissection. Eur J Vasc Endovasc Surg.

[2] Marui A., Mochizuki T., Mitsui N., Koyama T., Kimura F., Horibe M. (1999). Toward the best treatment for uncomplicated patients with type B acute aortic dissection: a consideration for sound surgical indication. Circulation.

[3] Hata M, Shiono M, Inoue T, Sezai A, Niino T, Negishi N (2003 Jun). Optimal treatment of type B acute aortic dissection: long-term medical follow up results. Ann Thorac Surg.

[4] Riambau V., Böckler D., Brunkwall J., Cao P., Chiesa R., Coppi G. (2017). Editor's Choice – management of descending thoracic aorta diseases: clinical practice guidelines of the European Society for Vascular Surgery (ESVS). Eur J Vasc Endovasc Surg.

[5] Aru R.G., Porez F., LE Houérou T., Palmier M., Gaudin A., Fabre D. (2025). Branched endovascular aortic repair of chronic post-dissection thoracoabdominal aortic aneurysms: an institutional experience on preoperative planning, intraoperative execution, and pitfalls. J Cardiovasc Surg (Torino).

[6] Tinelli G., Lescan M., Sica S., Piazza M., Bisdas T., Makaloski V. (2025). Inner branch off the shelf technology for chronic post-dissection thoraco-abdominal aneurysm with narrow true lumen: results of a European multicentre study. Eur J Vasc Endovasc Surg.

[7] Gallitto E., Faggioli G., Lodato M., Caputo S., Cappiello A., Di Leo A. (2025). T-branch by partial deployment technique in the endovascular repair of complex aortic and thoracoabdominal aneurysms with narrow or severe angulated para-visceral aorta. J Vasc Surg.

[8] Chait J., Tenorio E.R., Mendes B.C., Barbosa Lima G.B., Marcondes G.B., Wong J. (2022). Impact of gap distance between fenestration and aortic wall on target artery instability following fenestrated-branched endovascular aortic repair. J Vasc Surg.

[9] Kapalla M, Busch A, Lutz B, Nebelung H, Wolk S, Reeps C (2023 Jun 15). Single-center initial experience with inner-branch complex EVAR in 44 patients. Front Cardiovasc Med.

[10] Baghbani-Oskouei A., Savadi S., Mesnard T., Sulzer T., Mirza A.K., Baig S. (2023). Transcatheter electrosurgical septotomy technique for chronic postdissection aortic aneurysms. J Vasc Surg Cases Innov Tech.

